# Death Anxiety, Religious Doubt, and Depressive Symptoms across Race in Older Adults

**DOI:** 10.3390/ijerph16193645

**Published:** 2019-09-28

**Authors:** Kelcie D. Willis, Tamara Nelson, Oswaldo Moreno

**Affiliations:** 1 Department of Psychology, Virginia Commonwealth University, 806 West Franklin Street Box 842018, Richmond, VA 23284, USA; oamoreno@vcu.edu; 2 Department of Psychiatry and Human Behavior, Brown University, Providence, RI 02906, USA; tamara_nelson@brown.edu

**Keywords:** death anxiety, religious doubt, depression, race, aging

## Abstract

The purpose of this study is to investigate the direct and indirect relationships among death anxiety, religious doubt, and depressive symptoms in older adults. This study also investigates race as a moderator for these relationships. This study used data from the Religion, Aging, and Health Survey. Participants identified as Christian, identified as Black or White, lived in a non-institutionalized household within the U.S., were retired, and spoke English. Using PROCESS, results revealed that religious doubt partially mediated the relationship between death anxiety and depressive symptoms. Furthermore, moderated mediation models revealed that race moderated the relationship between religious doubt and depressive symptoms. Specifically, there was significant, positive relationship between religious doubt and depressive symptoms for participants who identified as Black but not White. Results highlight how religious doubt can influence depressive outcomes among the geriatric communities of color. Limitations and future directions are also discussed.

## 1. Introduction

As the number of older adults continues to rise in the United States [1], scholars continue to learn more about the nature of depression, which may present differently in this population compared to their younger counterparts [2]. Although older adults are less likely to be depressed [2], the consequences of depression in late adulthood can be quite substantial [3,4]. For example, depression among the geriatric population has been linked to decreased cognitive and physical functioning, as well as increased rates of mortality [3,4]. However, it is important to understand other potential, yet relevant factors that increase the risk for depression in older adulthood. 

Death anxiety, defined as the denial, fear, or negative beliefs one experiences when thinking about death and dying [5], may be one potential risk for depressive symptoms, as previous research has identified a connection between death anxiety and mental health [6]. Additionally, there are many factors that may affect an individual’s experience with death anxiety, including one’s religious beliefs [7]. Religious doubt, or the questioning of religious beliefs, teachings, or practices [8], may serve as the missing link that explains the relationship between death anxiety and depressive symptoms in older adults. Indeed, religious doubt has been shown to be positively associated with both depressive symptoms [8,9] and death anxiety [10,11]. In this study, we investigated the direct and indirect relationships among death anxiety, religious doubt, and depressive symptoms. Finally, we determined if race moderated this relationship, as previous research has found various aspects of religiosity, including religious doubt, to affect the mental health of White and Black older adults differently [8]. 

### 1.1. Death Anxiety and Depression

Death anxiety is an important variable in understanding geriatric mental health. While older adults might experience symptoms of generalized anxiety, such as worrying about their health or ability levels and somatic symptoms of stress [12], death anxiety refers to those symptoms of anxiety that are related to the end of life [13]. Although death anxiety tends to decrease with age, since older adults come to accept death more readily than their middle-aged counterparts [5,14], death anxiety is still a key factor to consider in geriatric populations for several reasons. First, death anxiety is associated with experience of illness and one’s quality of life [15,16]. Second, death anxiety has been associated with significant psychological problems, such as depression [5,6,17]. For example, in a meta-analysis by Fortner and Neimeyer [5], the authors reviewed 49 quantitative studies and found psychological distress, which included global measures of depressive symptoms, to be higher among individuals with more death anxiety. Similarly, Thorson and Powell [6] discovered that older adults who endorsed greater death anxiety also reported experiencing more depressive symptoms. Third, while research suggests a strong relationship between death anxiety and depression, much of this research is outdated [18,19,20]. Fourth, little is known about the factors that may be associated with the link between death anxiety and depressive symptoms. However, scholars have noted that individuals in the United States tend to place greater importance on their religion with age, and therefore, constructs under the umbrella of religiosity may be one variable influencing death anxiety’s effect on depressive symptoms in older adults [21]. Yet, to our knowledge, no study to date has investigated the effect of religious doubt as a mediator between death anxiety and depressive symptoms. 

### 1.2. Religious Doubt, Death Anxiety, and Depression

Religious doubt may impact the link between death anxiety and depressive symptoms. Religious doubt describes one’s struggles within their faith, religious teachings, and religious beliefs [22]. For instance, becoming skeptical about the divine in day-to-day decisions may be forms of religious doubt [8]. Some postulate that a healthy amount of doubt is normal for any religious individual [23]. Others recognize religious doubt as one manifestation of negative religious coping [24] and, thus, a potentially deleterious side to religious involvement. In fact, Krause and colleagues [25] describe religious doubt as an “unsettling” feeling similar to cognitive dissonance (i.e., when one experiences distress caused by holding two seemingly contradictory beliefs), where the religious person wrestles with two competing views of the world. Krause and colleagues [25] further explicate that there may be a stigma attached to questioning one’s faith from the faith community, which, in turn, might lead the religious person to experience feelings of guilt or shame. Indeed, the uncertainty that comes with religious doubt has been shown to correlate with both anxiety and depressive symptoms [9]; however, religious doubt is distinct from these variables as a complex construct of religiosity, largely understudied and not fully understood. Moreover, because religious doubt is related to psychological well-being, this variable may serve as an important link in the relationship between death anxiety and depressive symptoms.

Religious doubt is particularly relevant for the geriatric population: studies show that approximately 85% of Americans aged 65 or older identify their religious beliefs as “very important” or “somewhat important” to their daily lives [26]. The existing literature is consistent in that findings have demonstrated a positive and significant relationship between religious doubt and depressive symptoms [8,9]. More specifically, religious doubt tends to impact depressive symptoms via a multitude of pathways, such as by decreasing church attendance [27] and social support [8]. Age seems to be another important factor in the relationship between religious doubt and psychopathology, as Galek and colleagues [9], using quantitative data on 1629 adult participants (90.4% Caucasian, 4.7% Black/African American, 1% Hispanic/Latin American. 2% Asian, 2% other), found that the relationship attenuated as age increased. Though there seems to be a negative relationship between religious doubt and psychological well-being, no studies to date have considered the roles of other factors, such as death anxiety and race.

Religious doubt is also positively associated with death anxiety [10,11]. Ingram and Leitner [11], using a sample of 40 male Christian ministers, found a moderate-to-high positive correlation between death anxiety and religious doubt, as measured by the ambivalence subscale of the Dimension of Religious Ideology Scale, which is a subscale that captures those “skeptical” of their religious beliefs [28]. Furthermore, in a large sample (*n* = 634) of adults ranging from 18–89 years recruited from a university in the Mid-Atlantic, religious doubt was a significantly positively correlated to multiple domains of death anxiety, as measured by the Death Anxiety Inventory [10,29]. Investigators theorized that religious doubt influenced death anxiety by limiting participants’ worldview that could have otherwise buffered them from feeling uneasy about death. No study to date, however, has examined the link between death anxiety and religious doubt in older adults. Moreover, though previous research has demonstrated links between religiosity and both death anxiety and depression, the role of religious doubt as a potential mediator in the known relationship between death anxiety and depressive symptoms has not been investigated. 

### 1.3. Consideration of Race

The indirect relationship between death anxiety, religious doubt, and depression across race has not been examined. When considering these relationships, it is important to consider how these associations may vary across race for several reasons. First, studies have consistently noted that racial minority communities are more religious than their White counterparts [26,30]. Moreover, religious involvement has been found to provide social support and comfort as a means of coping with oppression and lack of resources [31,32]. Second, cross-comparison studies among older adults have found that buffering effects associated with religious involvement on both mental and physical well-being are stronger in Black participants. Specifically, Black older adults have endorsed greater improvements in physical health [33,34], subjective well-being [8], and depressive symptoms [35,36] as a function of their religious beliefs when compared to their White counterparts. These findings suggest that there might be a moderating effect of religious constructs, and religious constructs may be more salient in communities of color. However, less is known about religious doubt: this remains a gap in the psychological literature.

Religious doubt is a negative form of religious coping [24] and has been shown to exacerbate mental health concerns [25,37]. For instance, among both racial groups religious samples, religious doubt has been identified as a risk factor for poor psychological adjustment [37] and greater psychological distress [25]. While some theorize that religious doubt has a greater effect on those more deeply, religiously involved [38], such as Black communities, previous research has actually found religious doubt to have a more deleterious effect on White samples, a group that is typically less religiously involved [30]. For example, Krause’s [8] study of older Whites and African Americans revealed that religious doubt more heavily impacted the ability to form meaningful connections and relationships in the White, but not Black sample. Relatedly, in the same study, church attendance was more protective against the presence of religious doubt in White participants but not Black participants. These unexpected results may be due to the fact that Black communities express fewer religious doubts than their White counterparts [8]. Perhaps the infrequency of religious doubt in this community is the reason why researchers have found religious doubt to be more deleterious in White older adults. Consequently, a continued investigation of the relationship between race, religious doubt, and mental health is warranted.

### 1.4. Current Study 

**Figure 1:**
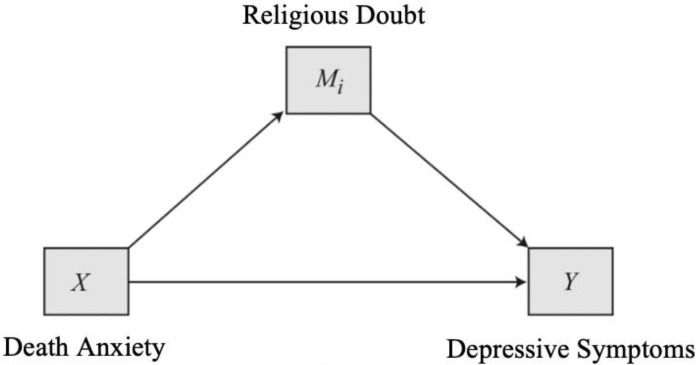
Conceptual diagram of the mediation analyses.

**Figure 2:**
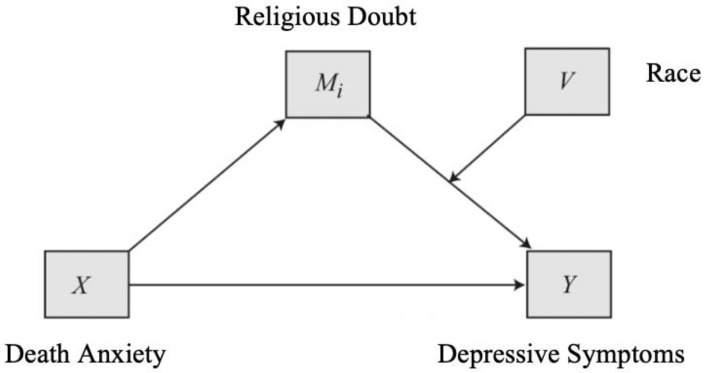
Conceptual diagram of the moderated mediation analysis.

Previous research has identified potential associations between death anxiety, depressive symptoms, and religious doubt but no study to date has considered how these three variables may fit together in one model. Moreover, there are heterogenous findings on how these relationships might differ across race [8,30]. As such, there were three major goals in the current study. First, we examined the relationship between death anxiety and depressive symptoms in a sample of religious older adults. Consistent with the results from an older meta-analysis [5], we hypothesized that there would be a positive, linear relationship between death anxiety and depressive symptoms in the sample. Secondly, we examined if and how religious doubt influenced the relationship between death anxiety and depressive symptoms (see in Figure 1). That is, we considered the extent to which religious doubt would mediate the relationship between death anxiety and depressive symptoms. We anticipated that increases in death anxiety would be associated with greater doubts about faith, which in turn, would be associated with increased depressive symptoms. Finally, we investigated if these mediational effects varied by race. Because researchers have found religiosity to differentially impact mental health by race [8], we expected moderated mediation in the link between religious doubt and depressive symptoms specifically (see in Figure 2). Consistent with Krause’s [8] findings, we expected there to be a stronger association between religious doubt and depressive symptoms in White older adults, due to the higher frequency of religious doubt, compared to their Black counterparts. 

## 2. Method

### Participants and Procedures

This study consists of a secondary analysis of data from the Religion, Aging, and Health Survey (RAHS) [39]. The RAHS is a national survey of older adults ages 66 and older who were recruited in 2004 and administered a battery of questionnaires that assessed religion, self-rated health, and psychological well-being. Participants completed specific measures about death anxiety, religious doubt, and depressive symptoms in their homes. The sampling method and details of the protocol for this study have been described in greater detail elsewhere [39]. Participants in this study (*N* = 993) identified as Christian, lived in a non-institutionalized household within the contiguous United States, were retired, and spoke fluent English. All participants in this study were religious and thus did not identify as atheist or agnostic. In terms of race, participants were 48.5% Black (*n* = 482) and 51.1% White (*n* = 511). With regard to sex, participants were 63% female (*n* = 626), and 37% male (*n* = 367). The mean age of the sample was 77.47 (*SD* = 6.14). In terms of annual family income 13.1% reported earning less than $10,000 per year (*n* = 130), 23.4% reported earning $10,000–$20,000 per year (*n* = 232), 15.4% reported earning $20,000–$30,000 per year (*n* = 153), 6.3% reported earning $30,000–$40,000 per year (*n* = 63), 6.0% reported earning $40,000–$60,000 per year (*n* = 60), 6.1% reported earning over $60,000 per year (*n* = 61). In terms of frequency of religious attendance, 20.6% endorsed never attending religious services (*n* = 205), 18.3% endorsed attending religious services a few times per year (*n* = 182), 10.1% endorsed attending religious services a few times per month (*n* = 100), 42.6% endorsed attending religious services weekly (*n* = 423), and 7.7% endorsed attending religious services multiple times per week (*n* = 76).

## 3. Measures

### 3.1. Demographics 

Participants provided information on their age, sex, marital status, religious affiliation, annual family income, religious attendance, and race.

### 3.2. Religious Doubt

Religious doubt was measured by the sum of five items that assess how often one feels uncertain about various aspects of their religious beliefs and/or practices [39]. Using a four-point Likert scale (1= never; 4 = always), participants rated items such as, “How often do you have doubts about your religious or spiritual beliefs?”, and “How often do you doubt that God is directly involved in your daily life?” This scale of religious doubt was appropriate as all participants identified as Christian. Higher scores indicate more uncertainty of religious beliefs and practices. The Cronbach’s alpha for this scale was 0.86. 

### 3.3. Death Anxiety

Four items from Neimeyer’s [40] handbook on death anxiety instrumentation was used to assess death anxiety. These items measure the degree to which participants feel troubled about death. Sample items include, “I find it hard to face up to the fact that I will die.”, and “I am disturbed by the shortness of life.” Responses ranged from 1 = strongly disagree and 4 = strongly agree and were summed together to create a total score where a higher score indicates increased anxiety surrounding death and dying. The Cronbach’s alpha for this scale was 0.89.

### 3.4. Depressive Symptoms

The Center for Epidemiological Studies Depression Inventory (CES-D) was used to measure depressive symptoms [41]. The CES-D is a self-administered questionnaire originally developed with 20 Likert scale items; however, a briefer eight-item version has been validated in older adults [42]. Items measure how often participants experience depressive symptoms such as decreased appetite or sleep. Responses ranged from 1 = rarely, 4 = most of the time. A total score was calculated by summing all eight responses and higher scores suggest increased distress. The Cronbach’s alpha for this scale was 0.88.

### 3.5. Rumination

Rumination was measured by the sum of four items that assess the extent to which individuals are unable to control their thoughts [39]. For example, sample items include “I have thoughts I cannot stop.”, and “I wish I could stop thinking about certain things.” Each item is scored on a Likert scale from 1 = strongly disagree and 4 = strongly agree. Higher scores indicate greater rumination. Cronbach’s alpha for this scale was 0.93.

### 3.6. Subjective Health

Participants self-rated their health status by one item developed by Krause [39]: “How would you rate your overall health at the present time?” Participants indicated if their health was excellent, good, fair, or poor on a four-point Likert scale. Higher scores indicated greater subjective health. 

### 3.7. Data Analytic Plan

First, we used the Missing Value Analysis (MVA) module in SPSS 26.0 to determine if variables were Missing Completely at Random (MCAR). Little’s MCAR [43] chi-square test is the most frequently used test for examining missing data. A non-significant *p*-value indicates that data are MCAR and missing values are ignorable and can be deleted. Next, we screened data for violation of statistical assumptions including linearity and normality (i.e., skewness, kurtosis, and outliers). Linearity was evaluated using bivariate scatter plots. Normality was evaluated using both graphical representations (i.e., histograms) as well as skewness and kurtosis. Any variables with skewness or kurtosis statistics beyond the +/−2.00 cutoff were transformed using square root, log 10, and inverse square root transformation for mild, moderate, and severe normality violations accordingly. Univariate outliers were examined by analyzing z-scores; cases were labeled outliers if the z-score was beyond three standard deviations from the mean [44]. Outliers were deleted if they comprise of more than 1–2% of the total sample [45]. Multivariate outliers were assessed using Mahalanobis distance (*D*^2^). 

We used analysis of variance (ANOVA) and crosstabs to examine differences in study variables by race. Given the significance of subjective health and rumination among older adults in its relationship to both anxiety and depressive symptoms, we included these variables as potential covariates. Bivariate Pearson correlations were examined to investigate the relationships among the variables of interest. All significant variables were controlled for in subsequent analyses. 

We used linear regression to examine the relationship between death anxiety and depressive symptoms. In addition, model 4 and model 14 of the Process Macro Version 3.4 [46] in SPSS 26.0 (SPSS Inc., Chicago, IL, USA) was used to investigate mediation (Figure 1) and moderated mediation models (Figure 2) respectively. Analyses were conducted using 5000 bootstrapping samples to produce 95% bias-corrected confidence intervals for indirect effects. Findings were significant if confidence intervals did not include zero. 

## 4. Results

### 4.1. Preliminary Results

The assumption of linearity was met via review of bivariate scatter plots. Furthermore, an inspection of histograms, skewness, and kurtosis indicated that the variables were normally distributed with the exception of religious doubt and depressive symptoms, which were positively skewed in the sample. Both of these variables were transformed using a log 10 transformation due to the moderate normality violation. Less than 1–2% of the total sample were identified as univariate or multivariate outliers; thus, the outliers were included in the current study. Finally, Little’s MCAR test revealed that less than 5% was missing. As data were missing completely at random, *X^2^* (9) = 4.00, *p* = 0.91, we did not include imputations for missing data. Thus, we removed cases with missing data.

Table 1 presents the weighted distribution of sociodemographic information and variables of interest stratified by race. Overall, there were significant differences between Black and White older adults with respect to marital status, annual family income, religious affiliation, religious attendance, and religious doubt. Black older adults were more likely to be separated/divorced or widowed, while white older adults were more likely to be married (*p* = 0.004). Black older adults were also more likely to report an annual family income of either under $10k or between $10–20k, while older White adults were more likely to report an annual family income of between $40–60k or over $80k and beyond (*p* < 0.001). In terms of religious affiliation, older Black adults were more like to identify as Protestant or Other, whereas older White adults were more like to identify as Catholic (*p* < 0.001). Additionally, Black older adults were more likely to attend religious services either “several times a month” or “several times a week”, and white older adults were more likely to “never” attend services (*p* < 0.001). Lastly, White older adults endorsed greater feelings of religious doubt (*p* = 0.003). We did not find any significant differences in rumination or subjective health between Black and White older adults. Thus, we excluded these variables from subsequent analyses. Table 2 provides the individual items of each measure and the correlation coefficients among study variables.

### 4.2. Direct Effects

We examined the relationship between death anxiety and depressive symptoms. When controlling for marital status, annual family income, religious affiliation, religious attendance, the results revealed that death anxiety was a significant predictor of depressive symptoms, *F*(5, 920) = 11.98, *β* = 0.15, *t* (920) = 4.58, *p* < 0.001, *R^2^* = 0.06. Moreover, an increase in death anxiety was associated with a greater endorsement of depressive symptoms. 

### 4.3. Indirect Effects

Death anxiety was expected to have a direct effect on depressive symptoms, as well as an indirect effect through religious doubt (Figure 1). When controlling for marital status, annual family income, religious affiliation, religious attendance, both the direct relationship from death anxiety to religious doubt (b = 0.01, *p* < 0.001) as well as the direct relationship from religious doubt to depressive symptoms (b = 0.09, *p* = 0.01) were significant. As participants endorsed greater death anxiety, they also reported more religious doubt; an increase in religious doubt was associated with more depressive symptoms. The indirect effect of death anxiety on depressive symptoms through religious doubt was also statistically significant (b = 0.001, 95% CI (0.0003, 0.002)), indicating a partial mediation: the direct relationship from death anxiety to depressive symptoms remained statistically significant when religious doubt was included in the model (b = 0.01, *p* < 0.001).

### 4.4. Moderated Indirect Effects

We used a conditional process model in order to determine whether the mediational effect differed by participants’ race (see Figure 2). When controlling for marital status, annual family income, religious affiliation, religious attendance, the overall model was significant, *F*(8, 889) = 7.42, *p* < 0.001, *R^2^* = 0.25. The direct effects from death anxiety to religious doubt and from race to depressive symptoms (b = −0.08, *p* = 0.01) were statistically significant (b = 0.01, *p* < 0.001), but the direct effect of religious doubt to depressive symptoms was not (b = −0.07, *p* = 0.33). The direct effect of death anxiety to depressive symptoms was also significant, b = 0.01, *p* < 0.01. Furthermore, the interaction between religious doubt and race was statistically significant (b = 0.10, *p* = 0.01), indicating that the direct effect of religious doubt to depressive symptoms was moderated by race. Specifically, for older adults who identify as Black, there was a positive indirect effect of death anxiety depressive symptoms through religious doubt (b = 0.001, 95% CI (0.001, 0.01)), while for those who identify as White, there was not (b = 0.001, 95% CI (−0.001, 0.001]). These findings indicate moderated mediation whereby an increase in death anxiety is associated with higher religious doubt, which predicted increased depressive symptoms only for those who identified as Black. 

## 5. Discussion

The purpose of the current study was to investigate the direct and indirect relationships among death anxiety, religious doubt, depressive symptoms, and race. Our initial findings are consistent with the overarching literature that suggests a significant and positive relationship between death anxiety and depressive symptoms in older adults [5,6]. Though results from previous studies [6] and meta-analyses [5] similarly suggest a significant association between death anxiety and depressive symptoms, much of this research is outdated [18,19,20]. Thus, the current study provides continued evidence that the presence of death anxiety may still be related to depressive symptomatology, though future studies in this area are still warranted due to the date of the current study’s data collection (i.e., early 2000s). Furthermore, while previous research suggests that levels of death anxiety actually decreases in older age [14], the results of the current study indicate that death anxiety can still have a detrimental effect in older adults. Because death anxiety was associated with increased depressive symptoms in our geriatric sample, future studies might consider predictors of death anxiety when studying this population. 

Additionally, the results of the current study suggest that increased death anxiety significantly predicted an increase in religious doubt. When one is experiencing fear about their death and perhaps the afterlife, it is intuitive that one might also be experiencing religious doubt, as religion may help to cope with existential issues [10]. In this way, this finding is consistent with previous research in adults that demonstrates a positive link between the two variables [10,11]; however, the current study is unique in that it is the first known study to examine the relationship between death anxiety and religious doubt in a sample of only older adults. Thus, it appears that the same existential processes that occur generally in adulthood also apply to older adults, a traditionally more religious demographic [26]. 

Next, increased religious doubt was, in turn, found to be related to greater depressive symptoms. Previous research on religious doubt has identified a similar pattern of results [8,9,27]. Yet, Fowler [23] questioned if religious doubt is normative and an important aspect of faith to some extent. Nonetheless, these findings revealed a positive association between religious doubt and depressive symptoms. Indeed, channels through which religious doubt influence mental health, such as by reducing church attendance [27] and limiting social support [8], continue to warrant investigation. These ideas are consistent with Koenig’s [47] seminal model of religiosity and health, which postulates that religious beliefs can create changes in one’s experience of positive emotions and social connections, which in turn, can lead one to experience increases in psychopathology, such as depressive symptoms. In this way, the current study illuminates the effect of religious beliefs in the form of religious doubt, which may impact a person’s mental health both positively and negatively. 

Additionally, our findings indicated that religious doubt mediated the relationship between death anxiety and depressive symptoms. Specifically, there was a direct relationship between death anxiety and endorsement of religious doubt, which in turn predicted depressive symptoms. This finding is consistent with both the general body of research where religiosity has been shown to influence mental health [48] as well as the specific body of research with religious doubt, where religious doubt has been found to be positively associated with both death anxiety [10,11] as well as depressive symptoms [8,9]. Krause [8] argued that this is due to the faith practitioner’s natural withdrawal from their religious community during periods of doubt. Perhaps the individual decides to isolate because of the guilt or shame that he or she feels from the stigma associated with questioning religious beliefs [25], yet further research is warranted. Krause and Ellison [49] found that individuals often have two responses when faced with religious doubt: they may either suppress doubts or seek spiritual support from fellow congregants when encountering doubt. The data of this study revealed that religious doubt had more of a deleterious health effect when the doubt was suppressed; on the other hand, seeking spiritual support during times of doubt had no significant impact on self-reported health. Moreover, additional research is needed to further elucidate the nuanced ways in which religious doubt might explain the relationship between death anxiety and depressive symptoms. 

When reviewing the literature on religiosity, death anxiety, and depressive symptoms, some have argued that more intrinsic proxies of religiosity are more strongly related to both death anxiety and depressive symptoms [48,50,51]. The current study provided evidence for a negative assessment of religiosity (i.e., religious doubt) as a mediator in the relationship between death anxiety and depressive symptoms, warranting further investigations on how aspects of religion may influence psychological well-being in a sample that identifies as highly religious. Though some have argued that religious doubt may not always be detrimental [23,49], studies have consistently found religious doubt to be correlated with negative mental health [8,9,25,37]. In this way, could other, negative aspects of religiosity (e.g., feeling punished by God) also partially explain the link between death anxiety and depressive symptoms? Religious doubt may also be a significant mediator due to the presence of two other negative, deficit measures in the model (i.e., death anxiety and depressive symptoms). This idea is consistent with previous research that has compared outcomes of both positive and negative religious coping: a study by Pargament, Smith, Koenig, and Perez [52] found that only negative religious coping (e.g., questioning God, reminiscent of religious doubt) was associated with depression in elderly hospitalized patients; positive religious coping (e.g., asking for forgiveness), on the other hand, was not found to be related to depression. The current study was the first to describe the link between death anxiety and depressive symptoms, through an investigation of one religious variable, religious doubt. Future studies might expand on this study by identifying other mediators under the umbrella of religiosity. 

Finally, these results indicated that the direct effect of religious doubt on depressive symptoms was moderated by race. Specifically, this relationship was significant for older adults who identified as Black, but not for those who identified as White. Thinking broadly, an array of studies that compare the effect of religiosity across racial groups has found a stronger link between religiosity and well-being (e.g., physical health, subjective well-being, and depressive symptoms) in Black older adults than their White counterparts [8,30,34]. Perhaps religious doubt also follows this pattern of results and is more potent in communities of color. Next, because religious doubt is associated with depressive symptoms through decreased church attendance and fewer meaningful relationships at church [8], perhaps religious doubt is associated with religious involvement. Research consistently demonstrates that Black older adults are typically more religiously involved than their White counterparts [26,30,53], which might explain why religious doubt is more harmful to this demographic. White older adults, who are typically less religiously involved when compared to their non-White older adult counterparts, might more effectively cope with their experience of doubt by reducing religious practices such as church attendance, thus aligning their beliefs with their behaviors and, in effect, reducing cognitive dissonance. It should be noted that even though Black older adults endorsed religious doubt less frequently than their White counterparts, these doubts seemed to have a stronger effect on their mental health as measured by depressive symptoms. In understanding this conundrum, it is evident that more research clarifying how religious doubt differentially impacts various racial groups is warranted. 

### Clinical Implications, Limitations, and Future Directions

The results of the current study illuminate the ways in which death anxiety is associated with depressive symptoms. Religious doubt was found to partially explain the link between death anxiety and depressive symptoms in a sample of older adults. Because religious doubt may serve as a risk for the geriatric population, professionals working with this population would do well to inquire about the religious beliefs and doubts of their older adult patients. In line with Krause’s [8] hypothesis that the experience of religious doubt might encourage individuals to withdraw from their religious communities, which in turn reduces one’s social support, professionals might encourage older adults who are religious to lean into their community and elicit spiritual support during this distressing time. Perhaps this approach would attenuate the relationship between religious doubt and depressive symptoms. Lastly, professionals working with this population may consider the cultural effect of religious doubt when working with older adult patients of color. Based on the results of this study, Black older adults may be at a greater risk for experiencing depressive symptoms as a result of their doubt than their White counterparts.

There are several limitations to this study worth noting. First, the cross-sectional study design is an important limitation. As in all cross-sectional studies, we make no causal inferences about the relationship between death anxiety, religious doubt, and depressive symptoms. This is important as we do not understand the potential long-term effects of religious doubt on psychological well-being. Future investigations might examine the extent to which religious doubt is normative, necessary, and an ultimately beneficial experience for the faith practitioner over time. Moreover, longitudinal designs would provide pertinent information about directionality and causality between death anxiety, religious doubt, and depressive symptoms. 

Second, the sample was fairly homogenous with regard to religious identity and race. All older adults identified as Christian and most identified as Protestant. Moreover, the data did not differentiate between mainline and Evangelical Protestants, and there is reason to believe that Evangelical Protestants would demonstrate greater depressive symptoms as a result of religious doubt. For example, Evangelical Protestants are more likely to discourage diverse or dissimilar beliefs from the larger group [54], which might ostracize an evangelical practitioner experiencing doubt to a greater extent. With this, the findings from this study may not generalize to other groups of older adults who do not identify as Christian and are not Black or White older adults. Future investigations might compare the results across Mainline and Evangelical Protestant denominations or investigate these relationships in other religious and ethnic communities (e.g., Latinx, Muslim, and Buddhist older adults). 

Finally, there was a low incidence of and little variation within the levels of religious doubt and depressive symptoms that were reported within the current sample. This might be due to social desirability. As all participants in the sample identified as Christians, in general, participants may have underreported religious doubt and depressive symptoms in this investigation. Future studies might consider other ways to measure religious doubt or other possible mediators such as negative religious coping [55]. Furthermore, using a structured interview to assess depression in older adults might be useful. It is important to note that religious doubt in the context of a specific religious group may be a different construct than having doubts about religion generally (e.g., those who identify as agnostic), and future studies might examine this nuance. Regardless, the current study expands upon the extant literature by identifying a significant mediating (i.e., religious doubt) and moderating (i.e., race) variable in the relationship between death anxiety and depressive symptoms in older adults. 

## 6. Conclusions

The findings of this study further elucidate the pathways in which older adults’ anxiety surrounding death impacts their depressive symptoms. Specifically, the results suggest that religious doubt can serve as mediator in this direct relationship, especially in Black communities. The information from this study provides clinicians a better understanding of predictors of depressive symptoms that may inform future clinical assessment and treatment.

## Figures and Tables

**Table 1:** Distribution of Sociodemographics, Death Anxiety, Depressive Symptoms, Religious Doubt, Rumination, and Subjective Health between Black and White Older Adults.

Variable	Overall Sample		Black		White		*p*-Value
	*N*	(%)	*N*	(%)	*N*	(%)	
Sex							0.49
Female	626	(63.0)	318	(66.0)	308	(60.3)	
Male	367	(37.0)	164	(34.0)	203	(39.7)	
Marital Status							0.004
Married	465	(46.8)	172	(35.7)	293	(57.3)	
Separated/Divorced	93	(9.4)	65	(12.5)	28	(5.5)	
Widowed	400	(40.3)	228	(47.3)	172	(33.7)	
Never Married	33	(3.3)	15	(3.1)	18	(3.5)	
Religious Affiliation							
Catholic	175	(17.6)	33	(6.8)	142	(27.8)	<0.001
Protestant	631	(63.5)	354	(73.4)	277	(54.2)	
Other	150	(15.1)	83	(17.2)	67	(13.1)	
Annual Family Income							
Less than 10k	130	(13.1)	108	(22.4)	22	(4.3)	<0.001
10–20k	232	(23.4)	135	(28.0)	97	(19.0)	
20–30k	153	(15.4)	58	(12.0)	95	(18.6)	
30–40k	63	(6.3)	21	(4.4)	42	(8.2)	
40–60k	60	(6.0)	11	(2.3)	49	(9.6)	
Over 60k	61	(6.1)	5	(1.0)	56	(10.9)	
Religious Attendance							<0.001
Never	205	(20.6)	53	(11.0)	152	(29.7)	
Few times a year	182	(18.3)	93	(19.2)	89	(17.4)	
Few times a month	100	(10.1)	71	(14.7)	29	(5.7)	
Weekly	423	(42.6)	215	(44.6)	208	(40.7)	
Several times a week	76	(7.7)	46	(9.5)	30	(5.9)	
Age (Mean, SD)	77.47	(6.16)	77.35	(6.23)	77.59	(6.09)	0.53
Death Anxiety (Mean, SD)	7.92	(2.68)	7.91	(2.77)	7.94	(2.60)	0.65
Religious Doubt (Mean, SD)	6.89	(2.99)	6.30	(2.50)	7.48	(3.30)	0.003
Depressive Symptoms (Mean, SD)	11.69	(4.78)	11.71	(4.70)	11.67	(4.85)	0.77
Rumination (Mean, SD)	9.45	(2.95)	9.60	(3.03)	9.32	(2.88)	0.49
Subjective Health (Mean, SD)	2.40	(0.92)	2.52	(0.86)	2.29	(0.95)	0.08

**Table 2:** Items and Pearson Correlation Coefficients of Study Variables.

Variable	1	2	3	4	5
1. Death Anxiety	1.00	0.17 **	0.13 **	0.19 **	0.04
I find it hard to face up to the fact that I will die.					
Thinking about death makes me feel uneasy.					
I do not feel prepared to face my own death.					
I am disturbed by the shortness of life.					
2. Religious Doubt		1.00	0.06	0.12 **	−0.02
How often do you have doubts about your religious or spiritual beliefs?					
How often do you have doubts about the things you’ve been taught in church?					
How often do you doubt whether solutions to your problems can be found in the Bible?					
How often do you doubt whether your prayers make a difference in your life?					
How often do you doubt that God is directly involved in your daily life?					
3. Depressive Symptoms			1.00	0.41 **	0.40 **
I felt I could not shake off the blues, even with the help of my family and friends.					
I felt depressed.					
I had crying spells.					
I felt sad.					
I did not feel like eating, my appetite was poor.					
I felt like everything I did was an effort.					
My sleep was restless.					
I could not get going.					
4. Rumination				1.00	0.19 **
I often have thoughts I try to avoid.					
There are thoughts that keep jumping into my head.					
I wish I could stop thinking about certain things.					
I have thoughts I cannot stop.					
5. Subjective Health					
How would you rate your overall health at the present time?					1.00

Note: ** *p* < 0.001.

## References

[1] Mather M., Jacobsen L.A., Pollard K.M. (2015). Population bulletin: Aging in the United States. Popul. Ref. Bur..

[2] Fiske A., Wetherell J.L., Gatz M. (2009). Depression in older adults. Ann. Rev. Clin. Psychol..

[3] Blazer D.G. (2013). Depression in late life: Review and commentary. J. Gerontol. Ser. A Biol. Sci. Med. Sci..

[4] Wu Z., Schimmele C.M., Chappell N.L. (2012). Aging and late-life depression. J. Aging Health.

[5] Fortner B.V., Neimeyer R.A. (1999). Death anxiety in older adults: A quantitative review. Death Stud..

[6] Thorson J.A., Powell F.C., Tomer A. (2000). Feelings about death in younger and older adults. Series in Death, Dying, and Bereavement. Death Attitudes and the Older Adult: Theories, Concepts, and Applications.

[7] Chan L.C., Yap C.C. (2009). Age, gender, and religiosity as related to death anxiety. Sunway Acad. J..

[8] Krause N. (2003). A preliminary assessment of race differences in the relationship between religious doubt and depressive symptoms. Rev. Relig. Res..

[9] Galek K., Krause N., Ellison C.G., Kudler T., Flannelly K.J. (2007). Religious doubt and mental health across the lifespan. J. Adult Dev..

[10] Henrie J., Patrick J.H. (2014). Religiousness, religious doubt, and death anxiety. Int. J. Aging Hum. Dev..

[11] Ingram B.J., Leitner L.M. (1989). Death threat, religiosity, and fear of death: A repertory grid investigation. Int. J. Pers. Constr. Psychol..

[12] Wolitzky-Taylor K.B., Castriotta N., Lenze E.J., Stanley M.A., Craske M.G. (2010). Anxiety disorders in older adults: A comprehensive review. Depress. Anxiety.

[13] Neimeyer R.A. (2015). Death Anxiety Handbook: Research, Instrumentation, and Application.

[14] Russac R.J., Gatliff C., Reece M., Spottswood D. (2007). Death anxiety across the adult years: An examination of age and gender effects. Death Stud..

[15] Bahrami N., Moradi M., Soleimani M.A., Kalantari Z., Hosseini F. (2013). Death anxiety and its relationship with quality of life in women with cancer. Iran J. Nurs..

[16] Sherman D.W., Norman R., McSherry C.B. (2010). A comparison of death anxiety and quality of life of patients with advanced cancer or AIDS and their family caregivers. J. Assoc. Nurses AIDS Care.

[17] Fortner B.V., Neimeyer R.A., Rybarczyk B., Tomer A. (2000). Correlates of death anxiety in older adults: A comprehensive review. Series in Death, Dying, and Bereavement. Death Attitudes and the Older Adult: Theories, Concepts, and Applications.

[18] Cataldo J.K. (1989). An Investigation of the Relationship of Hardiness and Death Attitudes to Depression in Older Persons in Skilled Nursing Facilities. Ph.D. Thesis.

[19] Templer D.I. (1971). Death anxiety as related to depression and health of retired persons. J. Gerontol..

[20] Williams A.K. (1985). Physical Illness and Depression: Changes over Time in Middle Aged and Elderly Persons. Ph.D. Thesis.

[21] Argue A., Johnson D.R., White L.K. (1999). Age and religiosity: Evidence from a three-wave panel analysis. J. Sci. Study Relig..

[22] Hunsberger B., McKenzie B., Pratt M., Pancer S.M., Lynn M.L., Moberg D.O. (1993). Religious doubt: A social psychological analysis. Research in the Social Scientific Study of Religion.

[23] Fowler J.W. (1976). Stages of Faith: The Psychology of Human Development and the Quest for Meaning.

[24] Pargament K.I., Koenig H.G., Perez L.M. (2000). The many methods of religious coping: Development and initial validation of the RCOPE. J. Clin. Psychol..

[25] Krause N., Ingersoll-Dayton B., Ellison C.G., Wulff K.M. (1999). Aging, religious doubt, and psychological well-being. Gerontologist.

[26] Pew Research Center Religious Landscape Study. Religion & Public Life. http://www.pewforum.org/religious-landscape-study/.

[27] Stark R., Finke R. (2000). Acts of Faith: Explaining the Human Side of Religion.

[28] Putney S., Middleton R. (1961). Dimensions and correlates of religious ideologies. Soc. Forces.

[29] Tomás-Sábado J., Gómez-Benito J. (2005). Construction and validation of the Death Anxiety Inventory (DAI). Eur. J. Psychol. Assess..

[30] Krause N. (2006). Exploring race and sex differences in church involvement during late life. Int. J. Psychol. Relig..

[31] Hope M.O., Assari S., Cole-Lewis Y.C., Caldwell C.H. (2017). Religious social support, discrimination, and psychiatric disorders among Black adolescents. Race Soc. Probl..

[32] Pargament K.I. (1997). The psychology of Religion and Coping.

[33] Ferraro K.F., Kim S. (2014). Health benefits of religion among Black and White older adults? Race, religiosity, and C-reactive protein. Soc. Sci. Med..

[34] Krause N. (2006). Exploring the stress-buffering effects of church-based and secular social support on self-rated health in late life. J. Gerontol. Ser. B Psychol. Sci. Soc. Sci..

[35] Krause N. (2003). Religious meaning and subjective well-being in late life. J. Gerontol. Ser. B Psychol. Sci. Soc. Sci..

[36] Taylor R.J., Chatters L.M., Abelson J.M. (2012). Religious involvement and DSM-IV 12 month and lifetime major depressive disorder among African Americans. J. Nerv. Ment. Dis..

[37] Cook S.W., Aten J.D., Moore M., Hook J.N., Davis D.E. (2013). Resource loss, religiousness, health, and posttraumatic growth following Hurricane Katrina. Ment. Health Relig. Cult..

[38] Krause N., Wulff K.M. (2004). Religious doubt and health: Exploring the potential dark side of religion. Sociol. Relig..

[39] Krause N. (2002). A comprehensive strategy for developing closed-ended survey items for use in studies of older adults. J. Gerontol. Ser. B Psychol. Sci. Soc. Sci..

[40] Neimeyer R.A. (1994). Feelings about Death Handbook: Research, Instrumentation, and Application.

[41] Radloff L.S. (1977). The CES-D scale a self-report depression scale for research in the general population. Appl. Psychol. Meas..

[42] Karim J., Weisz R., Bibi Z., ur Rehman S. (2015). Validation of the eight-item center for epidemiologic studies depression scale (CES-D) among older adults. Curr. Psychol..

[43] Little R.J. (1988). A test of missing completely at random for multivariate data with missing values. J. Am. Stat. Assoc..

[44] Hair J.F., Black W.C., Babin B.J., Anderson R.E., Tatham R.L. (2010). SEM: An introduction. Multivariate Data Analysis: A Global Perspective.

[45] Cohen J., Cohen P., West S.G., Aiken L.S. (2003). Applied Multiple Regression/Correlation Analysis for the Behavioral Sciences.

[46] Hayes A.F. (2017). Introduction to Mediation, Moderation, and Conditional Process Analysis: A Regression-Based Approach.

[47] Koenig H.G. (2012). Religion, spirituality, and health: The research and clinical implications. ISRN Psychiatry.

[48] Ronneberg C.R., Miller E.A., Dugan E., Porell F. (2014). The protective effects of religiosity on depression: A 2-year prospective study. Gerontologist.

[49] Krause N., Ellison C.G. (2009). The doubting process: A longitudinal study of the precipitants and consequences of religious doubt in older adults. J. Sci. Study Relig..

[50] Cicirelli V.G. (2002). Fear of death in older adults: Predictions from terror management theory. J. Gerontol. Ser. B Psychol. Sci. Soc. Sci..

[51] Wen Y.H. (2010). Religiosity and death anxiety. J. Hum. Resour. Adult Learn..

[52] Pargament K.I., Smith B.W., Koenig H.G., Perez L. (1998). Patterns of positive and negative religious coping with major life stressors. J. Sci. Study Relig..

[53] Wood J.B., Parham I.A. (1990). Coping with perceived burden: Ethnic and cultural issues in Alzheimer’s family caregiving. J. Appl. Gerontol..

[54] Kelley D. (1972). Why Conservative Churches Are Growing.

[55] Pargament K., Feuille M., Burdzy D. (2011). The Brief RCOPE: Current psychometric status of a short measure of religious coping. Religions.

